# Analysis of outcome indicators in clinical trials related to feeding intolerance in ICU patients receiving enteral nutrition feeding

**DOI:** 10.3389/fnut.2025.1666339

**Published:** 2025-09-15

**Authors:** Chaokai He, Xiaorong Li, Fan Li, Ying Xia, Kunrong Yu

**Affiliations:** ^1^Division of Pulmonary and Critical Care Medicine, Department of Internal Medicine, Peking Union Medical College Hospital, Chinese Academy of Medical Sciences and Peking Union Medical College, Beijing, China; ^2^Department of Family Medicine and Division of GlM, Department of Internal Medicine, Peking Union Medical College Hospital, Chinese Academy of Medical Sciences and Peking Union Medical College, Beijing, China; ^3^Department of Emergence, Peking Union Medical College Hospital, Chinese Academy of Medical Sciences and Peking Union Medical College, Beijing, China; ^4^Department of Internal Medicine, Peking Union Medical College Hospital, Chinese Academy of Medical Sciences and Peking Union Medical College, Beijing, China

**Keywords:** ICU, enteral nutrition, feeding intolerance, outcome indicators, core outcome set, clinical trials

## Abstract

**Purpose:**

The aim of this study was to analyze the outcome indicators of clinical trials related to feeding intolerance in patients receiving enteral nutrition feeding in ICUs published in the past 10 years, and to provide data support for the construction of a core outcome set for clinical trials on feeding intolerance in ICU patients.

**Methods:**

The databases of Cochrane Library, Pubmed, Embase, SinoMed, Wanfang Data and China Knowledge Network (CNKI) were searched using a combination of free and subject terms. The time limit was from January 2013 to September 2023. Literature screening and data extraction were carried out independently by two researchers strictly according to the inclusion and exclusion criteria, and in case of disagreement, the decision was discussed by a third party.

**Results:**

A total of 52 papers reporting 138 different outcome indicators were included in this study. Indicators were categorized into 8 domains based on their functional characteristics. In descending order of frequency of reporting, they were symptoms and signs (82.7%), physical and chemical tests (75%), indicators related to nutritional support (63.5%), safety events (59.6%), long-term prognosis (34.6%), economic assessment (21.2%), functional status (5.8%), and satisfaction (3.8%). The top 10 most frequently reported outcome indicators were diarrhea, vomiting, bloating, gastric remnants, reflux, aspiration, gastric retention, mortality, albumin and constipation. The main problems with the indicators included a lack of systematicity, clinical utility, and standardization of reporting, as well as inconsistency in the time point of measurement and misuse of the indicators.

**Conclusion:**

The lack of core outcome sets had led to significant variability and non-standardization in the outcome indicators reported by Enteral Feeding Intolerance clinical studies. Enteral Feeding Intolerance is an important factor affecting the prognosis of critically ill patients, and the outcome indicators of its clinical studies need to be standardized. It is recommended that a core outcome set for Enteral Feeding Intolerance be constructed to standardize the reporting of outcome indicators in future studies, reduce inter-study heterogeneity, and improve the utility of study results and the quality of clinical decision-making.

## 1 Introduction

Enteral nutritional support in the Intensive Care Unit (ICU) is an important part of the treatment of critically ill patients; however, the occurrence of Enteral Feeding Intolerance (EFI) has become a serious challenge, affecting the effectiveness of nutritional support and the prognosis of patients. EFI is commonly used to describe intolerance to enteral feeding due to manifestations of gastrointestinal dysfunction such as vomiting, gastric retention, and diarrhoea from any clinical cause (1). Studies have shown that the incidence of EFI is very high in critically ill patients, up to 10.95% in the first 7 days of ICU hospitalization (2), and is closely associated with poor clinical prognosis, such as prolonged mechanical ventilation, increased duration of vasoactive drug support, and increased mortality (3, 4). Furthermore, either persistence or recurrence of EFI in critically ill patients predicts an incremental risk of adverse outcomes (5). A study of COVID-19 critically ill patients noted a 56% incidence of EFIs in these patients, often presenting with symptoms such as massive gastric retention, abdominal distension, and vomiting, and was associated with an increased risk of multiorgan system complications such as cardiac, renal, hepatic, and hematological, leading to longer ICU stays, total length of hospital stay, and higher in-hospital mortality rates in patients with EFIs (6). Therefore, identification, prevention, and management of EFI are key to improving nutritional support and overall prognosis of critically ill patients. However, there is currently no reliable, validated, and widely recognized and used objective method to measure and assess EFI in critically ill patients (3), which seriously hampers the pace of development of its diagnostic practice and clinical research. The choice of endpoints to be measured and reported in EFI-related clinical trials poses a great challenge to researchers.

Domestic and international studies have found that outcome indicators reported in clinical trials in health-related fields generally suffer from selective reporting, high heterogeneity of indicator measurements, and impracticality of indicators, and even some crucial outcomes may be underestimated or completely ignored (7–10). Core Outcome Sets (COS) are the smallest set of outcome indicators that are agreed upon and should be reported consistently when conducting clinical trials and other health-related studies (11). COS play a crucial role in research in the health field, especially in improving the quality of reported outcome indicators in clinical trials, reducing study bias and strengthening the clinical evidence base. COS can well address the problems of selective reporting and high inter-study heterogeneity in outcome assessment, thus improving the quality of clinical decision-making and the utility of study results (12, 13). Therefore, this study aimed to analyze the systematic evaluation of outcome indicators reported in clinical trials of EFI in critically ill patients, thereby laying the foundation for clarifying and unifying the core set of indicators of EFI, in order to promote more effective and standardized development of research in related fields, and to provide strong evidence support for optimizing feeding intolerance intervention strategies in critically ill patients. In future studies, the Delphi method and consensus meetings will be employed to establish a core outcome set for EFI. This approach aims to minimize the heterogeneity in outcome reporting across subsequent studies, facilitate the synthesis of clinical research findings into robust, high-quality evidence, and ultimately enhance clinical decision-making and care in the management of EFI. The study protocol is registered with COMET (Core Outcome Measures in Effectiveness Trials) (registration number: 2799, https://comet-initiative.org/Studies/Details/2799) and is open access.

## 2 Methods

### 2.1 Inclusion and exclusion criteria

#### 2.1.1 Inclusion criteria

(1) Study type: all clinical trials of enteral nutrition support for feeding intolerance in critically ill patients, with the language limited to English and Chinese; (2) Patient type: adult patients (age ≥ 18 years) receiving enteral nutrition support in the intensive care unit and experiencing feeding intolerance, with no restriction on the type of patient’s disease, duration of the disease, mode of enteral nutrition support, gender, ethnicity and geographic location; (3) Intervention measures: Intervention programs such as pharmacological or non-pharmacological alone or in combination were used in the observation group, and control measures were not restricted; (4) Outcome indicators: all outcome indicators reported in the included studies were extracted.

#### 2.1.2 Exclusion criteria

(1) Patient type of minors aged <18 years; (2) Non-interventional studies; (3) Dissertations; (4) Review articles; (5) Conference abstracts; (6) Duplicate reports.

### 2.2 Literature search

(1) Search scope: the search period was from 01/2013 to 09/2023. Six databases were searched according to a combination of free and subject terms Cochrane Library, Pubmed, Embase, SinoMed, Wanfang Data and China National Knowledge Infrastructure (CNKI). (2) Search strategy: in order to avoid leakage, no excessive restrictions on the search conditions were imposed, and only Enteral Feeding Intolerance and Feeding Intolerance were searched for in English; and only Feeding Intolerance was searched for in Chinese. A combination of free-text and subject terms was used for the retrieval process. Taking the PubMed database as an example, the search query is (((“enteral nutrition”[MeSH Terms] OR (“enteral”[All Fields] AND “nutrition”[All Fields]) OR “enteral nutrition”[All Fields] OR (“enteral”[All Fields] AND “feeding”[All Fields]) OR “enteral feeding”[All Fields]) AND (“intolerabilities”[All Fields] OR “intolerability”[All Fields] OR “intolerable”[All Fields] OR “intolerably”[All Fields] OR “intolerance”[All Fields] OR “intolerances”[All Fields] OR “intolerant”[All Fields] OR “intolerants”[All Fields])) OR ((“feeding”[All Fields] OR “feedings”[All Fields] OR “feeds”[All Fields]) AND (“intolerabilities”[All Fields] OR “intolerability”[All Fields] OR “intolerable”[All Fields] OR “intolerably”[All Fields] OR “intolerance”[All Fields] OR “intolerances”[All Fields] OR “intolerant”[All Fields] OR “intolerants”[All Fields]))) AND (2013/1/1:2023/9/30[pdat]).

### 2.3 Literature screening and data extraction

Two evaluators read the titles and abstracts independently, and after excluding studies that clearly did not meet the inclusion criteria, studies that might meet the inclusion criteria were read in full text to evaluate whether they fully met the inclusion criteria, and then information was extracted from the included literature and cross-checked. Disagreements, if any, were resolved by discussion or consultation with a third party, and missing information was supplemented by contacting the original authors whenever possible. A pre-designed Excel spreadsheet was used to extract the data from the included literature, which included: (1) basic information of the study, including title, first author, year of publication, journal of publication, etc.; (2) baseline situation of the study subjects, including demographic characteristics such as the number of cases and age, as well as clinical characteristics such as the need for mechanical ventilation, enteral nutritional support modality, etc.; (3) interventions including details related to the drug intervention or non-pharmacological intervention and other relevant details as well as the duration of the intervention, etc.; (4) outcome indicators and their measurement methods and evaluation time points.

### 2.4 Risk of bias assessment

In this study, we employed the Cochrane Collaboration’s Risk of Bias Assessment Tool (RoB 2) (14) to evaluate the quality of included randomized controlled trials (RCTs) on enteral nutrition intolerance. The RoB 2 tool consists of five key domains: randomization process, deviation from the intended intervention, missing outcome data, outcome measurement, and selective reporting. Within each domain, studies were classified according to their risk of bias, with labels of “low risk” “some concerns” or “high risk” providing a comprehensive assessment of the literature quality and its potential bias.

## 3 Results

### 3.1 Literature screening process

**Figure 1:**
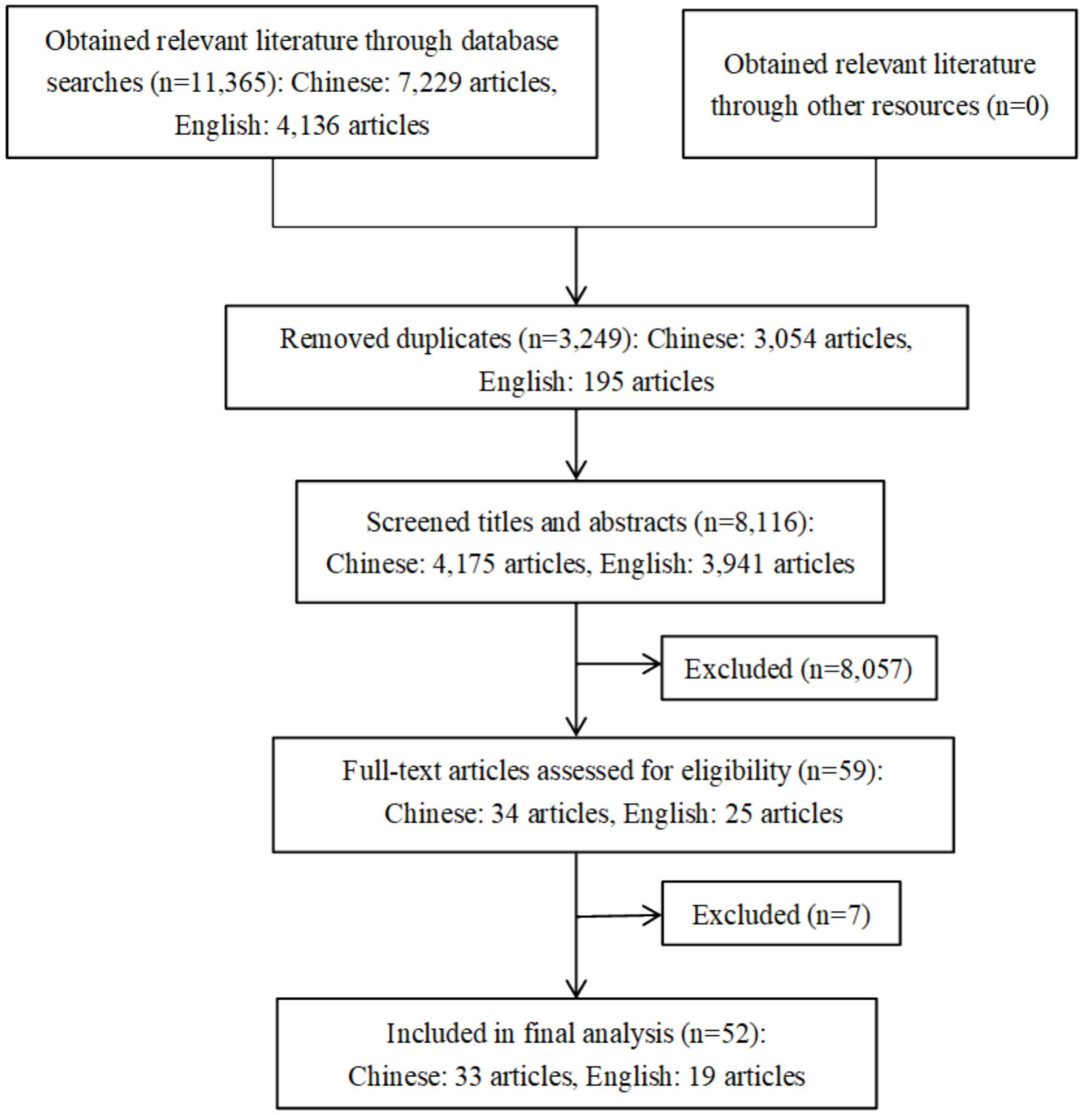
Flow diagram of included and excluded studies.

To evaluate the consistency between two independent reviewers during the literature screening stage, Cohen’s kappa value was calculated. The results demonstrated substantial agreement between the reviewers in the initial screening phase (kappa = 0.74), indicating a high level of reliability in the screening process. According to the search strategy initial inspection obtained 11,365 related literature, after deletion of duplicates remaining 8,116, through the title and abstract of the initial screening of the 59 literature for full-text reading, according to the inclusion and exclusion criteria, the exclusion of six observational studies and one duplicate report of the literature, and the final inclusion of 52 pieces of literature (15–66), of which 33 in Chinese, 19 in English. See Figure 1.

### 3.2 Basic characteristics of the included studies

#### 3.2.1 Sample size

A total of 5,463 patients were included in the 52 included studies, with an age span of 18 to 88 years, with a mean age of 51.78 ± 16.85 years. The sample size ranged from 13 to 792 cases, with a mean sample size of 105.06 ± 123.00 cases per study. More detailed study characteristics are provided in Supplementary File 1.

#### 3.2.2 Patient tracheal intubation

A total of 23 (23/52, 44%) studies reported respiratory support condition of tracheal intubation with ventilator assisted ventilation in critically ill patients, while 29 studies did not report respiratory support in critically ill patients.

#### 3.2.3 Patient nutritional support

A total of 37 (37/52, 71%) studies reported on patient nutritional support, of which 24 studies had nasogastric tube feeding, 7 studies had jejunal nutritional tube feeding, and 6 studies reported nutritional support by both nasogastric and jejunal nutritional tubes.

#### 3.2.4 Interventions

Interventions were divided into four main areas: pharmacological interventions, modification of feeding practices, integrated care programs, and transmission of medicinal techniques. Pharmacological interventions mainly involve the use of gastrointestinal power drugs, such as mosapride citrate dispersible tablets, neostigmine injection, domperidone tablets, metoclopramide hydrochloride injection, erythromycin enteric-coated capsules and so on; the adjustment of the feeding method mainly involves the use of jejunoileal nutritional tubes, adjusting the speed of feeding, or intermittent feeding and so on; the integrated nursing program mainly includes positional care, enteral nutritional supportive care, integrated pipeline care, gastric residual volume monitoring and other interventions. Residual volume monitoring and other interventions; traditional medicine interventions include herbal medicine, acupuncture, massage and acupoint injection.

#### 3.2.5 Course of treatment

Only 21 studies described the course of treatment, with a reporting rate of 12% (4/33) in the Chinese literature and 89% (17/19) in the English literature. The duration of treatment spanned from 3 days to 4 weeks, and the specific distribution was as follows: a total of 7 studies for 3 days of treatment, 4 studies for 5 days of treatment, 8 studies for 7 days of treatment, 1 study for 2 to 4 weeks of treatment, and 1 study for 4 weeks of treatment.

### 3.3 Risk of bias assessment results

This study utilized the Cochrane Collaboration’s Risk of Bias Assessment Tool (RoB 2) to evaluate the quality of the 52 randomized controlled trials (RCTs) on enteral nutrition intolerance included in the analysis. The results indicated that most studies performed well in terms of randomization processes and the management of missing outcome data, receiving a “low risk” rating. This suggests that these studies were relatively rigorous in randomization and data integrity. However, a small number of studies exhibited significant risk of bias, particularly in areas such as deviation from the intended intervention and selective reporting. Specifically, some studies demonstrated biases in the implementation of interventions, which could potentially affect the validity and reliability of the results. Furthermore, certain studies did not fully report all predefined outcomes, which compromised the comprehensiveness and transparency of the findings. Overall, most of the included studies demonstrated high methodological quality. More detailed results of the risk of bias assessment are provided in Supplementary File 2.

### 3.4 Outcome indicators

#### 3.4.1 Indicator domain

**Figure 2:**
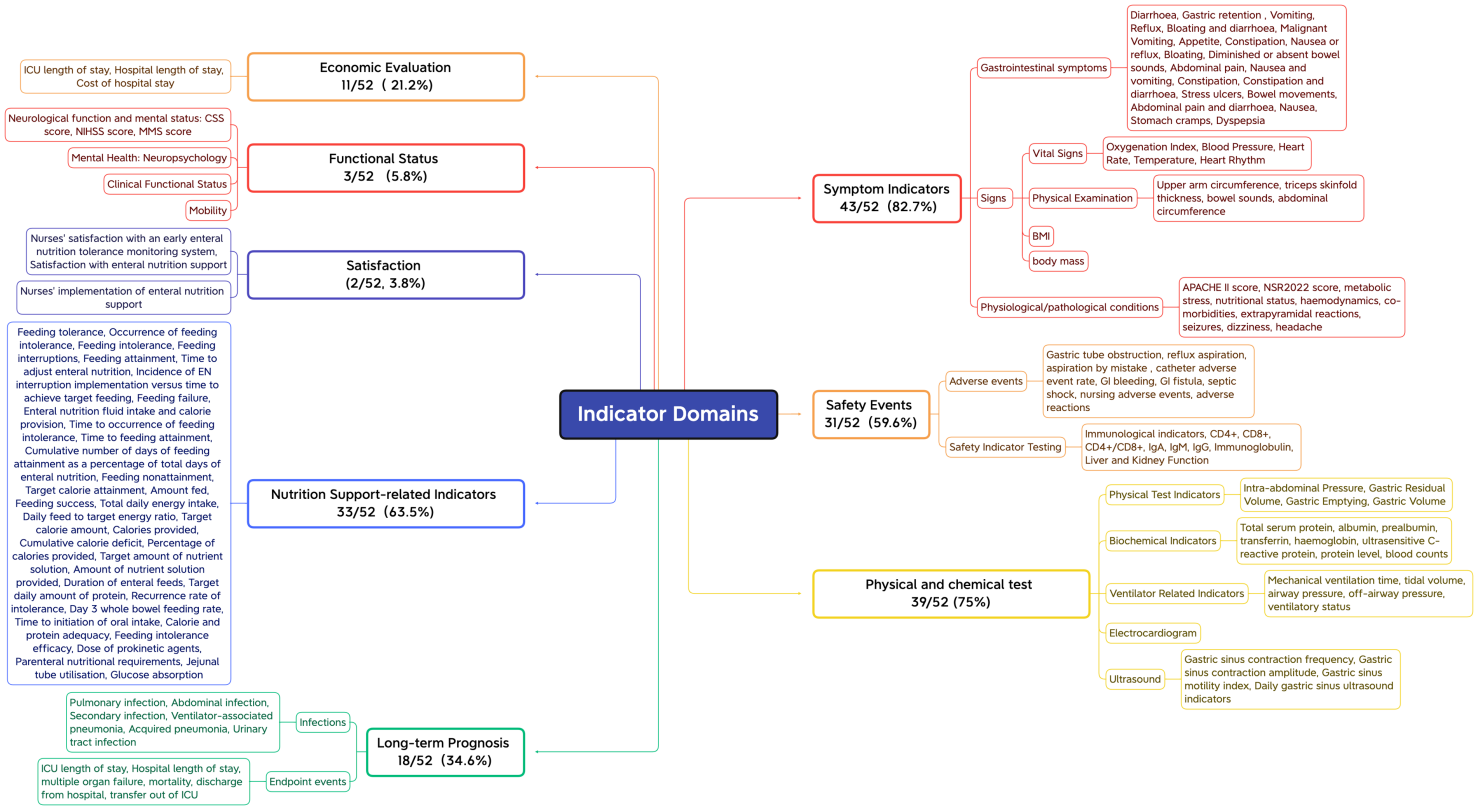
Outcome measures field.

First, outcome measures that assess the same concept but have different names or definitions are grouped together. Subsequently, based on the COMET initiative classification system (67) and the indicator classification method developed by Wang Keyi (68), the names of the grouped indicators are standardized and unified through expert panel discussions. This process ensures that the original meaning is preserved while providing a consistent description. Finally, the indicators are categorized into domains. The 52 included studies reported a total of 138 different outcome indicators, with a minimum of only 1 and a maximum of 17 outcome indicators reported by individual studies, and an average of approximately 8 outcome indicators reported per study. All reported outcome indicators were categorized into 8 domains based on the functional characteristics of the indicators, with symptomatic signs being reported in approximately 82.7% of the literature, the highest percentage, followed by physicochemical tests (75%), indicators related to nutritional support (63.5%), safety events (59.6%), long-term prognosis (34.6%), economic assessment (21.2%), functional status (5.8%) and satisfaction (3.8%). See Figure 2.

#### 3.4.2 Indicator frequency

Of the 52 papers included, the top 21 reported frequencies were, in order Abdominal distension, Vomiting, Abdominal bloating, Gastric residual volume, Gastric retention.

**Figure 3:**
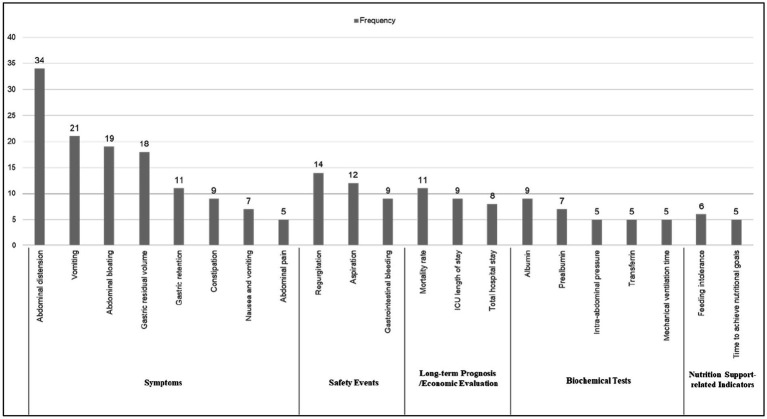
Top 21 outcome indicators reporting rate.

**Figure 4:**
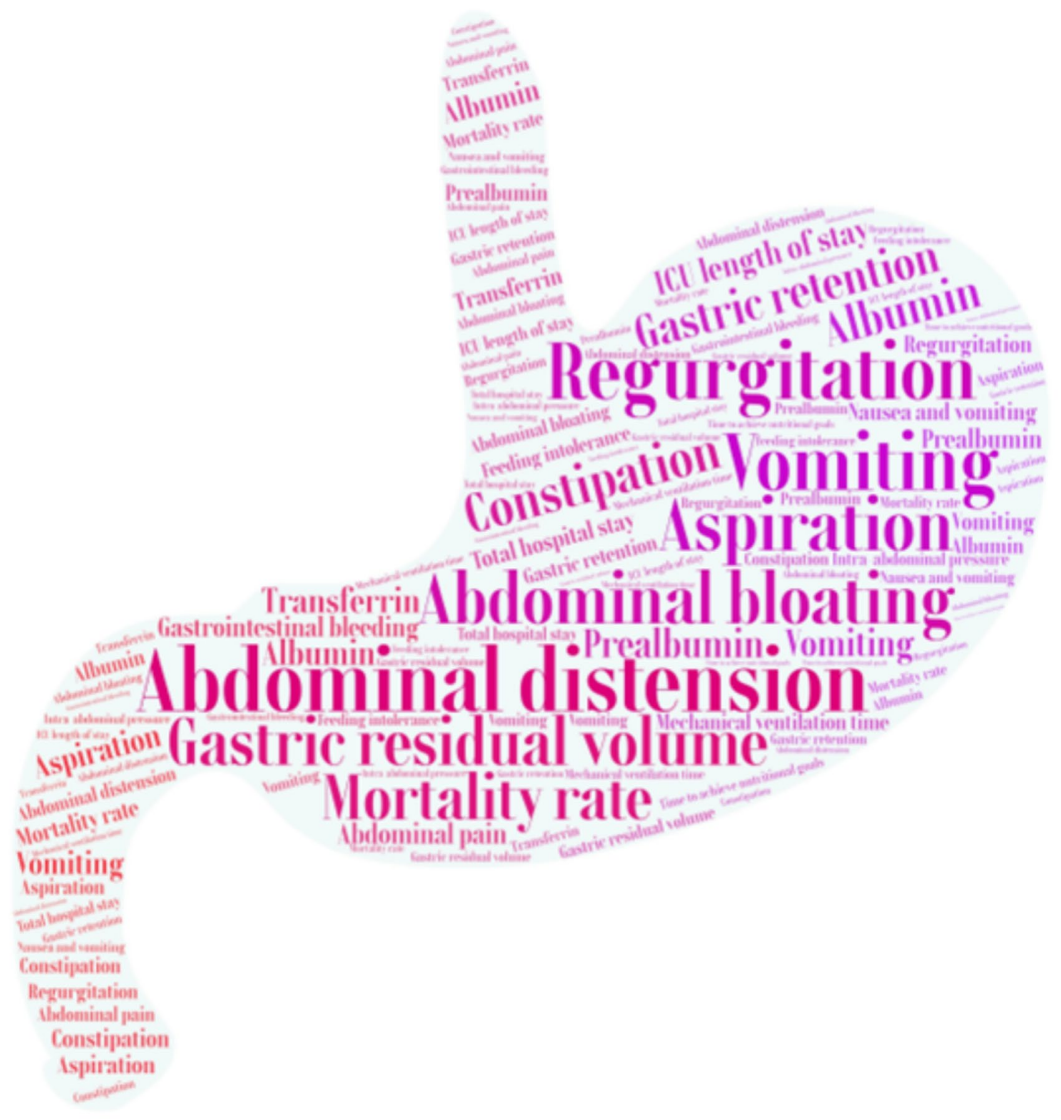
Word cloud of the top 21 outcome indicators.

Constipation, Nausea and vomiting, Abdominal pain, Regurgitation, Aspiration, Gastrointestinal bleeding, Mortality rate, ICU length of stay, Total hospital stay, Albumin, Prealbumin, Intra-abdominal pressure, Transferrin, Mechanical ventilation time, Feeding intolerance, Time to achieve nutritional goals. These indicators span six distinct areas, with the largest proportion falling under symptoms (8 indicators, 38.1%), followed by biochemical tests (5 indicators, 23.8%), safety events (3 indicators, 14.3%), long-term prognosis (3 indicators, 14.3%), economic evaluation (2 indicators, 9.5%), and nutrition support-related indicators (2 indicators, 9.5%). See Figure 3 for details. In addition, Figure 4 shows the word cloud of the top 21 outcome indicators, where the size of each word reflects its frequency in the included studies. The most frequently reported outcome indicators, such as “Abdominal distension” “Vomiting “and “Abdominal bloating “are displayed in larger fonts, highlighting their prominent presence in the literature. The word cloud visually emphasizes the most commonly studied indicators related to feeding intolerance in critically ill patients, making it easier to identify the key clinical parameters that are consistently reported across various studies.

#### 3.4.3 Indicator combinations

A total of 50 indicator combination types were reported in the 52 papers included in this study, with 10 papers reporting 5 outcome indicators, which ranked first in terms of frequency, followed by 7 and 10 indicators in 7 papers each. Only 3 literatures reported exactly the same indicator combination type.

#### 3.4.4 Indicator measurement time points

**Figure 5:**
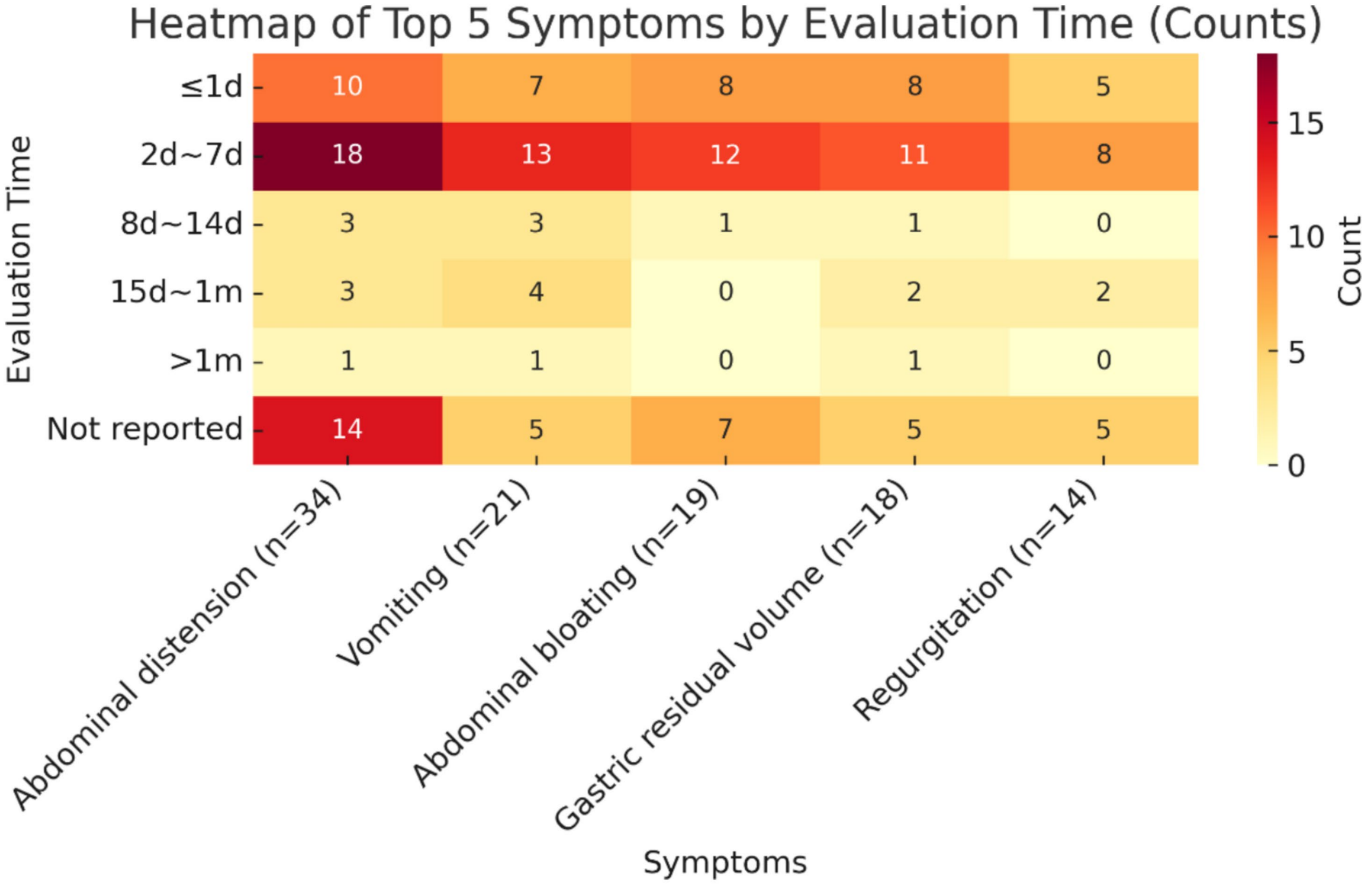
Heatmap of top 5 symptoms by evaluation time.

The validity indicators that ranked in the top 5 in terms of frequency of reporting were selected to summarize their measurement time points, and the results are shown below. The measurement time points of all five indicators were concentrated in the time period of 2d ~ 7d, which accounted for the highest proportion of all measurement time points, namely diarrhoea (18/34, 52.9%), vomiting (13/21, 61.9%), bloating (12/19, 63.2%), gastric remnant (11/18, 61.1%), and reflux (8/14, 57.1%), followed by the time period of ≤1d. In addition, the percentage of studies that did not report the time point of measurement was also relatively high, exceeding 20% in all cases, with a high percentage of 41.2% not reporting the time point of measurement for the indicator diarrhoea. See Figure 5 for details.

## 4 Discussion

In this study, for the first time, we conducted a comprehensive and integrated analysis of outcome indicators reported by clinical trials related to feeding intolerance in patients with enteral nutrition support in ICUs through a systematic evaluation approach. Our results found that clinical trial researchers had a large bias in the selection of outcome indicators, and the variability of outcome indicators measured and reported by different studies was extremely high, which was a prominent problem, mainly reflected in the following six aspects.

1. Lack of systematicity in outcome indicators. Currently, feeding intolerance, although lacking a comprehensive consensus definition, is classified in some of the more recognized evidence-based literature into three broad categories: large gastric remnants, gastrointestinal symptoms and enteral nutritional deficiencies (1, 3). However, existing clinical studies do not systematically report measures based on these broad categories, for example, some studies report only two or three of a series of gastrointestinal symptoms such as diarrhoea, bloating, vomiting, reflux, bleeding or constipation, or even only a single symptom (27, 28, 31, 49). Such one-sided selective reporting of an outcome indicator would make it difficult to objectively reflect the true clinical effectiveness of feeding intolerance interventions. Of course, this situation may be related to the unclear definition of feeding intolerance.
2. Lack of clinical utility of outcome indicators. Among the 138 indicators reported in the included literature, except for the categories of gastrointestinal symptoms and nutritional status, which were directly related to feeding intolerance, there were a large number of intermediate indicators that were used to assess the clinical efficacy of the interventions, such as dizziness, headache, neuropsychological, and mobility (17, 19, 29). In addition, some of the self-developed indicators, such as the occurrence of feeding intolerance and performance by nurses, are general in concept, lack uniform evaluation criteria and are too subjective (37, 51, 64). These outcome indicators cannot directly and objectively reflect the intervention effect of the clinical study on feeding intolerance, and their clinical utility is not high.
3. Lack of standardization in indicator reporting. It is mainly manifested in the unclear expression of indicator names, arbitrary splitting or combining, ambiguity and other aspects. For example, some studies used gastric residual volume to indicate gastric emptying, while some studies described it as gastric retention; a combination of indicators for two different symptoms was reported, such as nausea and vomiting, abdominal distension and constipation, constipation and diarrhoea, abdominal pain and diarrhoea, and regurgitation and miscarriage of bowel movements; in addition, the indicator of defecation can indicate both diarrhoea and constipation, but it was used to indicate the number of bowel movements in the original study.
4. Lack of consistency in the group of evaluation indicators. Fifty different combinations of efficacy evaluation indicators appeared in the 53 papers included, and the combinations of indicator groups spanned a wide range of arbitrariness, with the number of combinations ranging from a single one to a maximum of 17. Only 3 papers simultaneously reported the indicator combination type of enteral nutrition tolerance, aspiration, nausea and vomiting, diarrhoea and abdominal pain, and mortality.
5. Lack of uniformity in the time point of indicator measurement. For the same indicator, the measurement time points for outcome measures vary across different studies, and these differences increase the heterogeneity of the research results. For instance, the indicator of gastric residual volume was reported at up to 12 different time points. Denise Schulz (69) also observed in a systematic review that many studies exhibit significant variability in the timing of outcome assessments. Some studies report multiple, heterogeneous time points for the same indicator, which considerably reduces the comparability of results across studies (70). The heterogeneity in outcome measurement time points may have a substantial impact on evidence synthesis and future meta-analyses. Therefore, when conducting meta-analyses, researchers must account for this heterogeneity and may need to mitigate its impact on the synthesized results through stratification or sensitivity analysis of time points. Furthermore, to enhance the reliability and consistency of evidence synthesis, future studies should adopt standardized outcome time points whenever possible and clearly specify the measurement time points in their reports.
6. Misuse of outcome indicators. Some studies have misused and misapplied outcome indicators, such as dyspepsia, which suffers from inversion of cause and effect and should not be used as an evaluation indicator for intervention outcomes. In addition, indicators such as gastric tube obstruction, incidence of catheterized adverse events, and urinary tract infections are not directly related or even irrelevant to feeding intolerance and should not be used as outcome evaluation indicators.

In addition to evaluating and analyzing the problems of reporting outcome indicators in the original literature, this study will screen a pool of core indicator entries based on the quality and frequency of use of the indicators to provide data support for the construction of the COS Initial Correspondence List for Clinical Trials of Feeding Intolerance in Patients with Enteral Nutritional Support in ICUs.

## 5 Limitations and perspectives

This study only included intervention studies published in the last decade, and there are some limitations in the representativeness of the sample. Regarding the evaluation of indicators, this study did not further analyze the indicator measurement tools and specific methods, while the unification of measurement tools and methods also plays a very important role in the consistency of outcome indicators. Future studies should further expand the type of literature and do more in-depth evaluation and analysis on indicator measurement tools and methods. Additionally, while this review provides a comprehensive summary of the outcome measures reported in recent clinical studies on EFI, we have not proposed a unified definition of EFI. We acknowledge the heterogeneity and ambiguity surrounding the definitions of FI across various studies. Future research should focus on bridging this definitional gap and advancing the development of a consensus definition and a core set of outcome measures for EFI.

## 6 Summary

The unclear definition of feeding intolerance makes the evaluation of its clinical diagnosis and intervention effect complex and ambiguous. The outcome indicators reported in clinical studies related to feeding intolerance in patients with enteral nutritional support in ICUs mainly suffer from five major problems, such as lack of systematicity, impracticality in clinical practice, lack of standardization in presentation, arbitrary combination of indicator groups, and a wide span of measurement time points. These problems increase the heterogeneity of results among similar studies, making it difficult to facilitate the integration of evidence and reducing the value and practical significance of the findings for use in guiding clinical practice. Therefore, a consensus set of core indicators of feeding intolerance in patients with enteral nutritional support in ICUs needs to be constructed to improve the quality of clinical studies in related fields and reduce unnecessary research waste.

## Data Availability

The raw data supporting the conclusions of this article will be made available by the authors, without undue reservation.

## References

[1] Reintam Blaser A Malbrain ML Starkopf J Fruhwald S Jakob SM De Waele J. Gastrointestinal function in intensive care patients: terminology, definitions and management. Recommendations of the ESICM working group on abdominal problems. Intensive Care Med. (2012) 38:384–94. doi: 10.1007/s00134-011-2459-y, PMID: 22310869 PMC3286505

[2] Yu K Guo N Zhang D Xia Y Meng Y Weng L. Prevalence and risk factors of enteral nutrition intolerance in intensive care unit patients: a retrospective study. Chin Med J. (2022) 135:1814–20. doi: 10.1097/CM9.0000000000001974, PMID: 35833658 PMC9521784

[3] Reintam Blaser A Deane AM Preiser JC Arabi YM Jakob SM. Enteral feeding intolerance: updates in definitions and pathophysiology. Nutr Clin Pract. (2021) 36:40–9. doi: 10.1002/ncp.10599, PMID: 33242218

[4] Heyland DK Ortiz A Stoppe C Patel JJ Yeh DD Dukes G. Incidence, risk factors, and clinical consequence of enteral feeding intolerance in the mechanically ventilated critically ill: an analysis of a multicenter, multiyear database. Crit Care Med. (2021) 49:49–59. doi: 10.1097/CCM.0000000000004712, PMID: 33148950

[5] Hu B Sun R Wu A Ni Y Liu J Guo F. Prognostic value of prolonged feeding intolerance in predicting all-cause mortality in critically ill patients: a multicenter, prospective, observational study. JPEN J Parenter Enteral Nutr. (2020) 44:855–65. doi: 10.1002/jpen.1693, PMID: 31429970

[6] Liu R Paz M Siraj L Boyd T Salamone S Lite TLV. Feeding intolerance in critically ill patients with COVID-19. Clin Nutr. (2022) 41:3069–76. doi: 10.1016/j.clnu.2021.03.033, PMID: 33934924 PMC8007186

[7] Smith SM Wallace E Salisbury C Sasseville M Bayliss E Fortin M. A Core outcome set for multimorbidity research (COSmm). Ann Fam Med. (2018) 16:132–8. doi: 10.1370/afm.2178, PMID: 29531104 PMC5847351

[8] Gargon E Gurung B Medley N Altman DG Blazeby JM Clarke M. Choosing important health outcomes for comparative effectiveness research: a systematic review. PLoS One. (2014) 9:e99111. doi: 10.1371/journal.pone.0099111, PMID: 24932522 PMC4059640

[9] Kirkham JJ Davis K Altman DG Blazeby JM Clarke M Tunis S. Core outcome set-STAndards for development: the COS-STAD recommendations. PLoS Med. (2017) 14:e1002447. doi: 10.1371/journal.pmed.1002447, PMID: 29145404 PMC5689835

[10] He CK Liu CX Wang H Zhang JH. Analysis of outcome indicators of randomised controlled trials of Chinese medicine for breast cancer. Tianjin Tradit Chin Med. (2021) 38:1299–304. doi: 10.11656/j.issn.1672-1519.2021.10.16

[11] Kirkham JJ Gorst S Altman DG Blazeby J Clarke M Devane D. COS-STAR: a reporting guideline for studies developing core outcome sets (protocol). Trials. (2015) 16:373. doi: 10.1186/s13063-015-0913-9, PMID: 26297658 PMC4546355

[12] Webbe J Sinha I Gale C. Core Outcome Sets. Arch Dis Child Educ Pract Ed. (2018) 103:163–6. doi: 10.1136/archdischild-2016-312117, PMID: 28667046

[13] Kirkham JJ Williamson P. Core outcome sets in medical research. BMJ Med. (2022) 1:e000284. doi: 10.1136/bmjmed-2022-000284, PMID: 36936568 PMC9951367

[14] Sterne JAC Savović J Page MJ Elbers RG Blencowe NS Boutron I. RoB 2: a revised tool for assessing risk of bias in randomised trials. BMJ (Clinical research ed). (2019) 366:l4898. doi: 10.1136/bmj.l4898, PMID: 31462531

[15] Nasiri M Farsi Z Ahangari M Dadgari F. Comparison of intermittent and bolus enteral feeding methods on enteral feeding intolerance of patients with Sepsis: a triple-blind controlled trial in intensive care units. Middle East J Dig Dis. (2017) 9:218–27. doi: 10.15171/mejdd.2017.77, PMID: 29255580 PMC5726335

[16] Heyland DK van Zanten A Grau-Carmona T van Zanten ARH Evans D Beishuizen A. A multicenter, randomized, double-blind study of ulimorelin and metoclopramide in the treatment of critically ill patients with enteral feeding intolerance: PROMOTE trial. Intensive Care Med. (2019) 45:647–56. doi: 10.1007/s00134-019-05593-2, PMID: 31062046 PMC9121863

[17] Chapman MJ Jones KL Almansa C Barnes CN Nguyen D Deane AM. Blinded, double-dummy, parallel-group, phase 2a randomized clinical trial to evaluate the efficacy and safety of a highly selective 5-Hydroxytryptamine type 4 receptor agonist in critically ill patients with enteral feeding intolerance. JPEN J Parenter Enteral Nutr. (2021) 45:115–24. doi: 10.1002/jpen.1732, PMID: 31990087 PMC7891369

[18] Deng LX Lan C Zhang LN Dun T Yang S Qing Y. The effects of abdominal-based early progressive mobilisation on gastric motility in endotracheally intubated intensive care patients: a randomised controlled trial. Intensive Crit Care Nurs. (2022) 71:103232. doi: 10.1016/j.iccn.2022.103232 35397977

[19] Malekolkottab M Khalili H Mohammadi M Ramezani M Nourian A. Metoclopramide as intermittent and continuous infusions in critically ill patients: a pilot randomized clinical trial. J Comp Eff Res. (2017) 6:127–36. doi: 10.2217/cer-2016-0067, PMID: 28114798

[20] Reddy S Bailey M Beasley R Bellomo R Mackle D Psirides A. Effect of saline 0.9% or plasma-Lyte 148 therapy on feeding intolerance in patients receiving nasogastric enteral nutrition. Crit Care Resusc. (2016) 18:198–e6. PMID: 27604334. doi: 10.1016/S1441-2772(23)00946-8, PMID: 27604334

[21] Elmokadem EM El B R Bassiouny AM Hanna MG Darweesh E Sabri NA. The efficacy and safety of itopride in feeding intolerance of critically ill patients receiving enteral nutrition: a randomized, double-blind study. BMC Gastroenterol. (2021) 21:126. doi: 10.1186/s12876-021-01712-w 33740892 PMC7976729

[22] Zhang W Zhou W Kong Y Li Q Huang X Zhao B. The effect of abdominal massage on enteral nutrition tolerance in patients on mechanical ventilation: a randomized controlled study. Intensive Crit Care Nurs. (2023) 75:103371. doi: 10.1016/j.iccn.2022.103371, PMID: 36528462

[23] Makkar JK Gauli B Jain K Jain D Batra YK. Comparison of erythromycin versus metoclopramide for gastric feeding intolerance in patients with traumatic brain injury: a randomized double-blind study. Saudi J Anaesth. (2016) 10:308–13. doi: 10.4103/1658-354X.174902, PMID: 27375386 PMC4916815

[24] Charoensareerat T Bhurayanontachai R Sitaruno S Navasakulpong A Boonpeng A Lerkiatbundit S. Efficacy and safety of enteral erythromycin Estolate in combination with intravenous metoclopramide vs intravenous metoclopramide monotherapy in mechanically ventilated patients with enteral feeding intolerance: a randomized, double-blind, controlled pilot study. J Parenter Enter Nutr. (2021) 45:1309–18. doi: 10.1002/jpen.2013, PMID: 32895971

[25] Ozen N Tosun N Yamanel L Altintas ND Kilciler G Ozen V. Evaluation of the effect on patient parameters of not monitoring gastric residual volume in intensive care patients on a mechanical ventilator receiving enteral feeding: a randomized clinical trial. J Crit Care. (2016) 33:137–44. doi: 10.1016/j.jcrc.2016.01.028, PMID: 26948254

[26] Qiu C Chen C Zhang W Kou Q Wu S Zhou L. Fat-modified enteral formula improves feeding tolerance in critically ill patients: a multicenter, single-blind, randomized controlled trial. J Parenter Enter Nutr. (2017) 41:785–95. doi: 10.1177/0148607115601858, PMID: 26350918

[27] Dickerson RN Corley CE Holmes WL Byerly S Filiberto DM Fischer PE. Gastric feeding intolerance in critically ill patients during sustained pharmacologic neuromuscular blockade. Nutr Clin Pract. (2023) 38:350–9. doi: 10.1002/ncp.10911, PMID: 36156827

[28] Shaikh N Nainthramveetil MM Nawaz S Hassan J Shible AA Karic E. Optimal dose and duration of enteral erythromycin as a prokinetic: a surgical intensive care experience. Qatar Medical Journal. (2021) 2020, 1–11. doi: 10.5339/qmj.2020.36, PMID: 33447536 PMC7802089

[29] Vijayaraghavan R Maiwall R Arora V Choudhary A Benjamin J Aggarwal P. Reversal of feed intolerance by Prokinetics improves survival in critically ill cirrhosis patients. Dig Dis Sci. (2022) 67:4223–33. doi: 10.1007/s10620-021-07185-x, PMID: 34392492 PMC8364303

[30] Ben-Arie E Wei TH Chen HC Huang TC Ho WC Chang CM. Digestion-specific acupuncture effect on feeding intolerance in critically ill post-operative Oral and Hypopharyngeal Cancer patients: a single-blind randomized control trial. Nutrients. (2021) 13, 1–15. doi: 10.3390/nu13062110, PMID: 34205461 PMC8234819

[31] Oshvandi K Dehvan F Falahinia G Taher A Soltanian A Sadeghi-Hedayat S. The effects of nasogastric feeding at different intervals on feeding intolerance in ICU patients: a single-blind randomized controlled trial. Fam Med Prim Care Rev. (2020) 22:140–5. doi: 10.5114/fmpcr.2020.95322

[32] Chapman MJ Deane AM O'Connor SL Nguyen NQ Fraser RJ Richards DB. The effect of camicinal (GSK962040), a motilin agonist, on gastric emptying and glucose absorption in feed-intolerant critically ill patients: a randomized, blinded, placebo-controlled, clinical trial. Crit Care. (2016) 20:232 2016 Aug 1. doi: 10.1186/s13054-016-1420-4, PMID: 27476581 PMC4967996

[33] Kooshki A Khazaei Z Zarghi A Rad M Mohammadi HG Tabaraie Y. Effects of fenugreek seed powder on enteral nutrition tolerance and clinical outcomes in critically ill patients: a randomized clinical trial. Biomed Res Ther. (2018) 5:2528–37. doi: 10.15419/bmrat.v5i7.462

[34] Li L. Comparative study of tolerance level of different enteral nutrition modalities in ICU patients. Chin J Pract Nurs. (2014) 30:44–5. doi: 10.3760/cma.j.issn.1672-7088.2014.05.015

[35] Luo L. Evaluation of nursing intervention for intolerance of enteral nutrition feeding in critically ill patients in ICU. Int J Nurs. (2014) 33:606–8. doi: 10.3760/cma.j.issn.1673-4351.2014.03.061

[36] Pan M Feng MX. Analysis of the effect of information-based enteral nutrition tolerance dynamic intervention applied to critically ill patients. Int J Med Health. (2021) 27:2773–6. doi: 10.3760/cma.j.issn.1007-1245.2021.17.036

[37] Gu RR. Preventive effect of immune-based enteral nutrition support on feeding intolerance in critically ill patients. Nutr Health Care Guide. (2018):280. doi: 10.3969/j.issn.1006-6845.2018.36.275

[38] Cao L Ye XH Zhang LN Li J Sun Y Tian D. Study on the correlation between stepped early bed-discharge activity and enteral nutrition tolerance in critically ill patients. Chin J Pract Nurs. (2018) 34:648–51. doi: 10.3760/cma.j.issn.1672-7088.2018.09.003

[39] Quan Y Zhang S Chen J Liu F Yao H. Construction and application of early enteral nutrition tolerance monitoring system for ICU patients. Chin J Nurs. (2022) 57:773–8. doi: 10.3761/j.issn.0254-1769.2022.07.001

[40] Yue J Chen R Aniwajiang Z. Clinical observation on the treatment of mechanically ventilated patients with feeding intolerance with Houpu exhaustion compound. China Trad Chin Medicine Modern Distance Educ. (2021) 19:52–4. doi: 10.3969/j.issn.1672-2779.2021.20.021

[41] Ding Z. Effects of different enteral nutrition infusion modes on nutritional tolerance in critically ill patients in ICU. Qilu Nursing Journal. (2017) 23:55–6. doi: 10.3969/j.issn.1006-7256.2017.14.026

[42] Li H Wang H Li Y Xu Y Qu R Huang Z. Effects of different angles of prone position on early oxygenation and enteral nutrition tolerance in ARDS patients. J Nurs. (2020) 35:5–8. doi: 10.3870/j.issn.1001-4152.2020.23.005

[43] Shao X Lin Z Li Y Yu H Ding J Jiang Z. Study on the effect of enteral nutrition semi-cured intermittent feeding on reducing enteral nutrition intolerance in critically ill patients. PLA Nursing Journal. (2020) 37:60–2. doi: 10.3969/j.issn.1008-9993.2020.01.015

[44] Hu H. The role of intra-abdominal pressure combined with gastric residual volume monitoring in the observation of early enteral nutrition tolerance in intensive care unit patients. Chin Foreign Med Res. (2019) 17:167–9. doi: 10.14033/j.cnki.cfmr.2019.20.075

[45] Wang C. Effects of different infusion rates of enteral nutrition on feeding intolerance in mechanically ventilated critically ill patients. Healthy Friends. (2021) 16:135–6.

[46] Yi Y. Effect of clustering strategy in improving early enteral nutrition tolerance in critically ill patients. Gansu Med. (2018) 37:1142–4.

[47] Li Y. Application study of quality nursing interventions on enteral nutrition tolerance and feeding compliance rate in patients with AECOPD undergoing invasive mechanical ventilation. Electron J Pract Clin Nurs. (2020) 5:143–52.

[48] Dai R Xie J Feng X. Correlation between stepped early bed mobility and enteral nutrition tolerance in critically ill patients. Healthy Friends. (2020):72.

[49] Li Y Shao X Jiang Z. Effectiveness of early enteral nutrition cluster feeding programme on nutritional calorie compliance and feeding intolerance in critically ill patients. Military Nursing. (2022) 39:41–4.

[50] Xiong Z Jiang H Xu X Li D. Clinical study on prevention and treatment of early enteral nutrition feeding intolerance in mechanically ventilated patients with sepsis by injection of astragalus injection at the foot-sanli acupoint. Modern J Integrative Med. (2022) 31:88–91. doi: 10.3969/j.issn.1008-8849.2022.01.019

[51] Gao T Liu J Huang L. Effects of Zhuang medicine thread point moxibustion on feeding tolerance and nutritional status of patients with acute gastrointestinal injury in sepsis. Chin Med Clin Res. (2020) 12:72–4.

[52] Su F Peng H. Intervention effect of healthcare co-operation strategy on reducing enteral nutrition feeding intolerance in ICU patients. World Digest Latest Med Inform. (2019) 19:89–90. doi: 10.19613/j.cnki.1671-3141.2019.88.054

[53] Lin B Xu L. Evidence-based management of feeding intolerance symptoms in critically ill nasogastric patients. J Nurs. (2019) 34:103–6. doi: 10.3870/j.issn.1001-4152.2019.16.103

[54] Li J Qiao Y. Analysis of the clinical value of comprehensive nursing intervention methods on enteral nutrition feeding intolerance in critically ill patients in ICU. Electron J Clin Med Liter. (2019) 6:146. doi: 10.16281/j.cnki.jocml.2019.56.125

[55] Zheng L Xu Z Jiang F. A study on the prevention and treatment of feeding intolerance in critically ill patients by meridian flow injection acupuncture. Chin Foreign Med Res. (2019) 17:24–6. doi: 10.14033/j.cnki.cfmr.2019.14.009

[56] Liu S Xie B Xu J Hu Y Fu X. Effects of different infusion rates of enteral nutrition on intra-abdominal pressure and feeding intolerance in mechanically ventilated patients in ICU. J Huzhou Normal College. (2019) 41:64–7. doi: 10.3969/j.issn.1009-1734.2019.04.011

[57] Wang H. Analysis of the clinical value of comprehensive nursing intervention methods on enteral nutrition feeding intolerance in critically ill patients in ICU. Electron J Pract Clin Nurs. (2018) 3:29–33.

[58] Liu Z Gao J Yang F Li M Meng L Xu Z. Influence and clinical effect of applying early post-pyloric feeding on enteral nutrition intolerance symptoms in critically ill patients. China Coal Industry Med J. (2022) 25:104–7. doi: 10.11723/mtgyyx1007-9564202201023

[59] Chen X Wei Q Zhang Y. Clinical effects of comprehensive nursing intervention methods on enteral nutrition feeding intolerance in critically ill patients in ICU. China Higher Med Educ. (2017) 10:135–6. doi: 10.3969/j.issn.1002-1701.2017.10.071

[60] Chen L Xue R Gao H. Application effect of continuous quality improvement in preventing enteral nutrition feeding intolerance in ICU patients. Contemp Nurses. (2014) 10:104–6.

[61] Lin F Zhang S Deng F. Effect of early enteral nutrition nursing programme on feeding intolerance in intensive care unit patients. Integrative Nurs Chin Western Med. (2023) 9:91–3. doi: 10.11997/nitcwm.202302025

[62] Hou H Yuan Y Zhang Y Zhang Q. Matching analysis of propensity for intolerance to enteral nutrition in 17 cases of mechanically ventilated critically ill patients treated with electroacupuncture. Liaoning J Chin Med. (2023) 50:201–5. doi: 10.13192/j.issn.1000-1719.2023.11.054

[63] Fang Y He H Zhang J Peng X Zhu J. Effect of prophylactic application of gastrointestinal stimulants on the occurrence of feeding intolerance in patients with severe craniocerebral injury. J Trauma Surg. (2022) 24:902–7. doi: 10.3969/j.issn.1009-4237.2022.12.005

[64] Wang S Xu S Wang A Ye X. Effect of enteral nutrition safe care programme on nutritional status and feeding intolerance in critically ill patients. Nurs Pract Res. (2022) 19:3549–53. doi: 10.3969/j.issn.1672-9676.2022.23.015

[65] Zhu J Li H Gong Y. Comparison of the effects of intermittent and continuous enteral nutrition on feeding intolerance in ICU patients with cerebral haemorrhage. Modern J Integrative Med. (2022) 31:3347–50. doi: 10.3969/j.issn.1008-8849.2022.23.027

[66] Li D Zhou H He W Wang H Xu Y. Preliminary study of acupuncture in improving the tolerance and feeding effect of transgastric feeding in critically ill patients. Parenter Enteral Nutr. (2020) 27:16–20. doi: 10.16151/j.1007-810x.2020.01.005

[67] Dodd S Clarke M Becker L Mavergames C Fish R Williamson PR. A taxonomy has been developed for outcomes in medical research to help improve knowledge discovery. J Clin Epidemiol. (2018) 96:84–92. doi: 10.1016/j.jclinepi.2017.12.020, PMID: 29288712 PMC5854263

[68] Wang K Jin X Wang H Zhang M Li N Pang W. Clinical study indicators analysis of traditional Chinese medicine treatment for unstable angina pectoris. Tianjin J Tradit Chin Med. (2020) 37:1150–5. doi: 10.11656/j.issn.1672-1519.2020.10.17

[69] Schulz D Gaeth C Jordan MC Herath SC Spering C Bieler D. Developing a core outcome set for acetabular fractures: a systematic review (part I). Syst Rev. (2025) 14:83. doi: 10.1186/s13643-025-02824-0, PMID: 40205445 PMC11983908

[70] Paul E George J Ward S Fitzgerald K Jones G Magana K. Assessing uptake of the core outcome set in randomized controlled trials for Parkinson’s disease: a systematic review. Ageing Res Rev. (2023) 91:102081. doi: 10.1016/j.arr.2023.102081, PMID: 37774933

