# Supplementary material for: Analysis of outcome indicators in clinical trials related to feeding intolerance in ICU patients receiving enteral nutrition feeding
Source: Front Nutr. 2025 Sep 15;12:1666339. doi: 10.3389/fnut.2025.1666339 (PMC12477000; doi:10.3389/fnut.2025.1666339)
Supplement: Supplementary file 1 [file Table_1.DOCX]

**Table 1. Study characteristics**

| **Serial Number** | **Included Study** | **Sample Size** | **Age** | **Diagnosis** | **ICU Type** | **Mechanical Ventilation (Yes/No/Not Described)** | **Gastric Tube / Jejunal Tube** | **Intervention** |
| --- | --- | --- | --- | --- | --- | --- | --- | --- |
| 1 | Li Li, 2014 | 77 | 37-67 | Intracerebral hemorrhage / Chronic obstructive pulmonary disease (COPD) with respiratory failure / Postoperative (surgical) / Post-cardiopulmonary resuscitation / Infectious toxic shock / Advanced cachexia of various causes | ICU | Not Described | Nasogastric tube / Jejunal tube | Gastrointestinal tube |
| 2 | Luo Liming, 2014 | 38 | 20-72 | Not Described | ICU | Not Described | Nasogastric tube | Comprehensive nursing care |
| 3 | Gu Rongrong, 2018 | 86 | 27-78 | Not Described | ICU | Not Described | Jejunal tube | Administration of immune-enhancing enteral nutrition formula |
| 4 | Pan Min, 2021 | 70 | 30-55 | Pulmonary infection / Septic shock / Myocardial infarction / Multiple trauma / Others | ICU | Not Described | Jejunal tube | Informatics-based dynamic intervention for enteral nutrition tolerance |
| 5 | Cao Lan, 2018 | 50 | 21-70 | Severe acute pancreatitis / Severe pneumonia / Intracranial aneurysm / Post-abdominal surgery / Acute respiratory distress syndrome (ARDS) | Department of Critical Care Medicine | Mechanical ventilation | Not Described | Stepwise early mobilization program out of bed |
| 6 | Quan Yinyin, 2022 | 120 | 37-59 | ICU patients | ICU | Not Described | Nasogastric tube / Jejunal tube | Use of ICU early enteral nutrition tolerance monitoring system |
| 7 | Yue Jinfang, 2021 | 120 | 53-69 | Intracerebral hemorrhage / Cerebral infarction / Severe pneumonia / Chronic obstructive pulmonary disease | ICU | Yes | Not Described | "Houpo Exhaustion Mixture (Ruiyang Pharmaceutical Co., Ltd., National Drug Approval No. Z20050563), 50 mL each time, twice daily, warmed and shaken before nasogastric feeding" |
| 8 | Ding Zhaohong, 2017 | 108 | 34-88 | ICU critically ill patients | ICU | Not Described | Nasogastric tube | Different enteral nutrition infusion methods: intermittent 24-hour pump infusion and continuous 24-hour pump infusion |
| 9 | Li Hao, 2022 | 45 | 44-67 | Acute respiratory distress syndrome (ARDS) | Department of Respiratory Critical Care Medicine | Yes | Nasogastric tube / Jejunal tube | Prone positioning at different angles: 0°, 30°, 45° |
| 10 | Shao Xiaoping, 2020 | 93 | 55-77 | Critically ill patients | ICU | Not Described | Jejunal tube | Semi-solid enteral nutrition intermittent feeding: addition of soluble dietary fiber (pectin) with intermittent feeding |
| 11 | Hu Huimin, 2019 | 100 | 31-78 | Emergency and critical patients | ICU | Not Described | Not Described | Combined monitoring of intra-abdominal pressure and gastric residual volume |
| 12 | Wang Cui, 2021 | 84 | 25-78 | Mechanically ventilated critically ill patients | ICU | Yes | Not Described | Different infusion speeds (initial rate 20 mL/h or 60 mL/h, then both groups gradually increased to 100 mL/h) |
| 13 | Yi Yuhua, 2018 | 460 | 17-79 | Severe patients | ICU, Neurology Intensive Care Unit, CCU (Coronary Care Unit) | Not Described | Nasogastric tube | Early enteral nutrition bundled nursing intervention |
| 14 | Li Yan, 2020 | 60 | 18-75 | Acute exacerbation of chronic obstructive pulmonary disease (AECOPD) | ICU | Not Described | Not Described | Implementation of high-quality nursing intervention: health education (videos, comics, brochures), early functional training, increased ward rounds, respiratory training, nutritional supplementation |
| 15 | Dai Ru, 2020 | 60 | 19-71 | Not Described | ICU | Not Described | Not Described | Stepwise early mobilization program out of bed |
| 16 | Hou Haikun, 2023 | 60 | 44-83 | Invasive mechanical ventilation patients | Intensive Care Unit (ICU) | Yes | Nasogastric tube / Jejunal tube | Electroacupuncture treatment |
| 17 | Liu Shanshan, 2019 | 120 | 59-70 | Patients receiving mechanical ventilation | ICU | Yes | Not Described | Different infusion rates |
| 18 | Wang Shengli, 2022 | 85 | 22-75 | Stroke / Severe traumatic brain injury / Cerebrovascular disease / Chronic obstructive pulmonary disease / Others | ICU | Not Described | Jejunal tube | Safety care plan |
| 19 | Lin Bixia, 2019 | 60 | 52-84 | Not Described | Neurology ICU | Not Described | Nasogastric tube | Evidence-based intervention (summary of evidence for managing feeding intolerance symptoms in critically ill nasogastric-fed patients) |
| 20 | Zhu Juan, 2022 | 118 | 53-70 | Intracerebral hemorrhage | ICU | Not Described | Not Described | Intermittent vs. continuous enteral nutrition |
| 21 | Liu Zhidan, 2022 | 79 | 20-70 | Critically ill patients | Intensive Care Unit (ICU) | Not Described | Jejunal tube | Early post-pyloric feeding |
| 22 | Su Fengjuan, 2019 | 100 | 20-78 | Not Described | ICU | Not Described | Nasogastric tube | Nurse–physician cooperation strategy |
| 23 | Fang Yuli, 2022 | 117 | 32-62 | Severe traumatic brain injury | ICU | Not Described | Nasogastric tube | Prophylactic use of gastrointestinal motility drugs: mosapride citrate dispersible tablets, neostigmine injection, domperidone tablets, metoclopramide hydrochloride injection, enteric-coated erythromycin capsules |
| 24 | Lin Fang, 2023 | 80 | 52-60 | Not Described | ICU | Not Described | Jejunal tube | · Early enteral nutrition nursing protocol |
| 25 | Li Yangyang, 2022 | 320 | ≥18 | Not Described | General ICU | Not Described | Not Described | Bundled early enteral nutrition feeding protocol: EN necessity assessment, safety assessment, caloric and protein requirements evaluation, feeding rate adjustment, gastrointestinal management strategies |
| 26 | Li Dan, 2020 | 40 | 38-78 | Not Described | ICU | Not Described | Nasogastric tube | Acupuncture treatment |
| 27 | Gao Tianye, 2020 | 80 | 38-70 | Sepsis with acute gastrointestinal injury | Department of Critical Care Medicine | Not Described | Not Described | Zhuang medicine medicinal thread moxibustion |
| 28 | Zheng Leilei, 2019 | 60 | 25-84 | Not Described | Department of Critical Care Medicine | Not Described | Nasogastric tube | Noon meridian time-based acupoint selection for acupuncture |
| 29 | Chen Xixiu, 2017 | 792 | 22-73 | Not Described | ICU | Not Described | Not Described | Comprehensive nursing care: medication administration, enteral nutrition support nursing |
| 30 | Li Juan, 2019 | 68 | 37-89 | Not Described | ICU | Not Described | Jejunal tube | "Comprehensive nursing care: position care, medication administration, enteral nutrition support nursing, comprehensive catheter care, other care: blood supply status, local skin" |
| 31 | Wang Hua, 2018 | 68 | 20-75 | Not Described | ICU | Not Described | Not Described | Comprehensive nursing care: integrated position care, integrated medication guidance, integrated enteral nutrition support nursing, comprehensive catheter care |
| 32 | Xiong Zhuowu, 2022 | 62 | 33-65 | Sepsis | ICU | Yes | Not Described | Acupoint injection of astragalus injection at Zusanli (ST36) |
| 33 | Chen Li, 2023 | 78 | 47-93 | Acute cerebrovascular disease / Digestive system diseases / Respiratory system diseases / Advanced malignancy / Severe traumatic brain injury / Others | ICU | Not Described | Nasogastric tube | "Continuous quality improvement" |
| 34 | Jeetinder Kaur Makkar, 2016 | 115 | 21-54 | Traumatic brain injury (TBI) | Intensive Care Unit | Yes | Nasal gastric tube | erythromycin (250 mg) vs metoclopramide (10 mg) |
| 35 | Taniya Charoensareerat, 2021 | 35 | 45-82 | Not Described | medical ICU and medical respiratory care unit | Yes | Nasogastric tube | enteral erythromycin estolate in combination with intravenous metoclopramide |
| 36 | Nurten Ozen, 2016 | 51 | ≥18 | Not Described | Intensive Care Unit | Yes | Not Described | GRV measured |
| 37 | Roland N. Dickerson, 2023 | 47 | 21-47 | Not Described | intensive care unit (TICU) | Yes | Nasogastric tube | initiation of a prokinetic agent |
| 38 | Nissar Shaikh, 2020 | 313 | 18-60 | Post-surgical/Post Traum/Spontaneous Subarachnoid hemorrhage/Intracerebral hemorrhage/Post Cardiac arrest | SICU /TICU | Yes | Nasal gastric tube | erythromycin in combination with metoclopramide |
| 39 | Morteza Nasiri, 2017 | 60 | 18-65 | patients with sepsis | ICUs | Not Described | Nasal gastric tube | nasogastric feeding at different intervals |
| 40 | Daren K. Heyland, 2019 | 120 | 40-74 | critically ill patient | ICU | Yes | Nasogastric tube | ulimorelin or metoclopramide |
| 41 | Chunfang Qiu, 2015 | 144 | 45-80 | Not Described | ICUs | Yes | Nasogastric tube, Jejunal tube | A Fat-Modified Enteral Formula |
| 42 | Marianne J. Chapman, 2021 | 13 | 18-85 | critically ill patient | ICU | Yes | Nasogastric tube, Jejunal tube | TAK-954 and metoclopramide |
| 43 | Lu-Xi Deng, 2022 | 83 | 34-68 | Not Described | ICU | Yes | Nasogastric tube | The abdominal-based early progressive mobilization program |
| 44 | Masoume Malekolkottab, 2017 | 40 | 40-80 | Baseline diseases :CV diseases /DM / Neuropsychiatric disease /CV diseases and DM /CV and neuropsychiatric diseases/DM and neuropsychiatric disease/Respiratory diseases / CV and respiratory diseases | ICU | Yes | Nasal gastric tube | metoclopramide either as intermittent infusion (10 mg every 6 hours) or continuous infusion (2 mg/h) |
| 45 | Sumeet Reddy, 2016 | 69 | 30-70 | critically ill patient | ICU | Yes | Nasal gastric tube | Plasma-Lyte 148 therapy |
| 46 | Eman Mohamed Elmokadem, 2021 | 76 | 31-53 | Surgical intervention/Trauma/CVS disorder/Respiratory disorder/Neurological disorder/Burn | ICU | Not Described | Nasal gastric tube | Itopride and Metoclopramide |
| 47 | Weiqin Zhang, 2023 | 74 | 18-75 | Intracerebral hemorrhage /Subarachnoid hemorrhage / Cerebral infarction / Diabetes mellitus / Hyperth/Gastrointestinal bleeding | intensive care unit (ICU) | Yes | Nasal gastric tube | abdominal massage |
| 48 | Khodayar Oshvandi, 2020 | 63 | 38-80 | Trauma/neurological problems/ Respiratory problems | intensive care units (ICUs) | Yes | Nasal gastric tube | nasogastric feeding at different intervals |
| 49 | Rajan Vijayaraghavan, 2022 | 83 | 32-60 | cirrhosis patients | intensive care units (ICU) | Not Described | Not Described | intravenous metoclopramide, erythromycin, or placebo |
| 50 | Eyal Ben-Arie, 2021 | 26 | 50-60 | Critically Ill Post-Operative Oral and Hypopharyngeal Cancer Patients | surgical and burn ICUs | Yes | Nasal gastric tube | Acupuncture |
| 51 | Marianne J. Chapman, 2016 | 33 | 18-85 | Trauma /Head injury /Respiratory failure /Sepsis /Other | intensive care unit (ICU) | Yes | Nasogastric tube | camicinal, a motilin agonist |
| 52 | Akram Kooshki, 2018 | 60 | 18-8 | Head trauma /Multi-organ trauma /Diabetes disease /Respiratory failure /Cardiac pathology /Neurological pathology /Digestive pathology /Glandular pathology/Infectious pathology | two intensive care unit (ICU) centers | Yes | Nasogastric tube | fenugreek seed powder |
