# Supplementary material for: Analysis of outcome indicators in clinical trials related to feeding intolerance in ICU patients receiving enteral nutrition feeding
Source: Front Nutr. 2025 Sep 15;12:1666339. doi: 10.3389/fnut.2025.1666339 (PMC12477000; doi:10.3389/fnut.2025.1666339)
Supplement: Supplementary file 2 [file Table_2.DOCX]

**Table2. Risk of Bias Assessment Results**

| **First Author** | **Publication Year** | **Randomization Process** | **Deviations from Intended Interventions** | **Missing Outcome Data** | **Measurement of Outcome** | **Selection of Reported Result** |
| --- | --- | --- | --- | --- | --- | --- |
| Yue Jinfang | 2021 | Low | Low | Low | Low | Low |
| Quan Yinyin | 2022 | Some concerns | Low | Low | Low | Low |
| Dai Ru | 2020 | Low | Low | Low | Low | Low |
| Ding Zhaohong | 2020 | Low | Low | Low | Low | Low |
| Li Hao | 2020 | Low | Low | Low | Low | Low |
| Shao Xiaoping | 2020 | Low | Low | Low | Low | Low |
| Hu Huimin | 2019 | Low | Low | Low | Low | Low |
| Wang Cui | 2021 | Low | Low | Low | Low | Low |
| Yi Yuhua | 2018 | Low | Low | Low | Low | Low |
| Li Yan | 2020 | Low | Low | Low | Low | Low |
| Hou Haikun | 2025 | Some concerns | Some concerns | Low | Low | Low |
| Liu Shanshan | 2024 | Low | Low | Low | Low | Low |
| Zhu Juan | 2023 | Some concerns | Some concerns | Low | Low | Low |
| Wang Shengli | 2024 | High | Some concerns | High | Low | Low |
| Chen Li | 2023 | Low | Some concerns | Low | Low | Low |
| Lin Bixia | 2023 | Some concerns | Some concerns | Low | Low | Low |
| Liu Zhidan | 2024 | Some concerns | Some concerns | Low | Low | Low |
| Su Fengjuan | 2023 | Low | Low | Low | Low | Low |
| Fang Yuli | 2024 | Low | Some concerns | Low | Low | Low |
| Lin Fang | 2023 | Some concerns | Some concerns | High | Low | Low |
| Li Yangyang | 2023 | Some concerns | Some concerns | Low | Low | Low |
| Li Dan | 2023 | Some concerns | Some concerns | Low | Low | Low |
| Gao Tianye | 2024 | Some concerns | Some concerns | Low | Low | Low |
| Zheng Leilei | 2023 | Low | Low | Low | Low | Low |
| Chen Xixiu | 2024 | Some concerns | Some concerns | Low | Low | Low |
| Li Juan | 2023 | Low | Some concerns | High | Low | Low |
| Wang Hua | 2023 | Some concerns | Some concerns | Low | Low | Low |
| Xiong Zhuowu | 2023 | Low | Some concerns | Low | Low | Low |
| Gu Rongrong | 2018 | Low | Some concerns | Low | Low | Low |
| Cao Lan | 2018 | Low | Low | Low | Low | Low |
| Pan Min | 2021 | Low | Some concerns | Low | Low | Low |
| Luo Liming | 2014 | Some concerns | Some concerns | Low | Low | Low |
| Zhang Lina | 2018 | Low | Some concerns | Low | Low | Low |
| Makkar JK | 2016 | Low | Some concerns | Low | Low | Low |
| Charoensareerat T | 2021 | Low | Low | Low | Low | Low |
| Ozen Nurten | 2016 | Some concerns | Some concerns | Low | Low | Low |
| Qiu Chunfang | 2015 | Low | Low | Low | Low | Low |
| Dickerson RN | 2023 | Low | Low | Low | Low | Low |
| Shaikh N | 2020 | Low | Some concerns | Low | Low | Low |
| Nasiri M | 2017 | Low | Low | Low | Low | Low |
| Heyland D | 2019 | Low | Some concerns | Low | Low | Low |
| Chapman M | 2021 | Low | Some concerns | Low | Low | Low |
| Deng L-X | 2022 | Low | Some concerns | Low | Low | Low |
| Elmokadem E | 2021 | Low | Some concerns | Low | Low | Low |
| Oshvandi K | 2020 | Low | Some concerns | Low | Low | Low |
| Malekolkottab M | 2017 | Low | Low | Low | Low | Low |
| Zhang W | 2023 | Low | Some concerns | Low | Low | Low |
| Reddy S | 2016 | Low | Low | Low | Low | Low |
| Vijayaraghavan R | 2022 | Low | Some concerns | Low | Low | Low |
| Ben-Arie E | 2021 | Low | Some concerns | Low | Low | Low |
| Chapman M | 2016 | Low | Low | Low | Low | Low |
| Kooshki A | 2018 | Low | Some concerns | Low | Low | Low |
